# The roles of Jumonji-type oxygenases in human disease

**DOI:** 10.2217/epi.13.79

**Published:** 2014-02

**Authors:** Catrine Johansson, Anthony Tumber, KaHing Che, Peter Cain, Radoslaw Nowak, Carina Gileadi, Udo Oppermann

**Affiliations:** 1 Structural Genomics Consortium, University of Oxford, Old Road Campus, Roosevelt Drive, Headington, OX3 7DQ, UK; 2 Botnar Research Center, NIHR Oxford Biomedical Research Unit, Nuffield Department of Orthopaedics, Rheumatology & Musculoskeletal Sciences, Oxford, OX3 7LD, UK; 3 Systems Approaches to Biomedical Sciences, Industrial Doctorate Center (SABS IDC) Oxford, UK

**Keywords:** chromatin modification, epigenetic, histone demethylation, Jumonji, lysine methylation, oxygenases

## Abstract

The iron- and 2-oxoglutarate-dependent oxygenases constitute a phylogenetically conserved class of enzymes that catalyze hydroxylation reactions in humans by acting on various types of substrates, including metabolic intermediates, amino acid residues in different proteins and various types of nucleic acids. The discovery of *jumonji* (Jmj), the founding member of a class of Jmj-type chromatin-modifying enzymes and transcriptional regulators, has culminated in the discovery of several branches of histone lysine demethylases, with essential functions in regulating the epigenetic landscape of the chromatin environment. This work has now been considerably expanded into other aspects of epigenetic biology and includes the discovery of enzymatic steps required for methyl-cytosine demethylation, as well as modification of RNA and ribosomal proteins. This overview aims to summarize the current knowledge on the human Jmj-type enzymes and their involvement in human pathological processes, including development, cancer, inflammation and metabolic diseases.

## Background

Several landmark discoveries have defined the Jumonji (Jmj) oxygenase protein family and provided important connections to chromatin and human biology. This family of iron (Fe^2+^)- and 2-oxoglutarate (2-OG)-dependent enzymes has its phylogenetic roots in prokaryotes, thus highlighting the functional versatility and critical importance of this protein class for life in an oxygen atmosphere [1]. Regarding their roles in chromatin biology, the first breakthrough discovery was a gene trap approach to identify novel factors involved in mouse embryonic development – this study defined the *jmj* gene (named *JARID2* in humans) as a novel class of transcriptional regulators. The gene was given the Japanese name *jumonji*, literally translated as ‘cruciform’, based on the cross-like shape found during neural groove development of *jmj* mutant mice [2,3]. Analysis of the domain arrangements and primary structure of this gene identified an ARID/Bright domain, found in many DNA-binding proteins, immediately suggesting chromatin association of the gene product, as well as novel, so-called Jmj N and C domains (JmjN and JmjC, respectively) [3,4]. Further bioinformatic analysis of the JmjC domain revealed this to be an evolutionarily conserved protein fold belonging to the cupin family [5].

## Mechanistic features of Jmj enzymes

**Figure 1:**
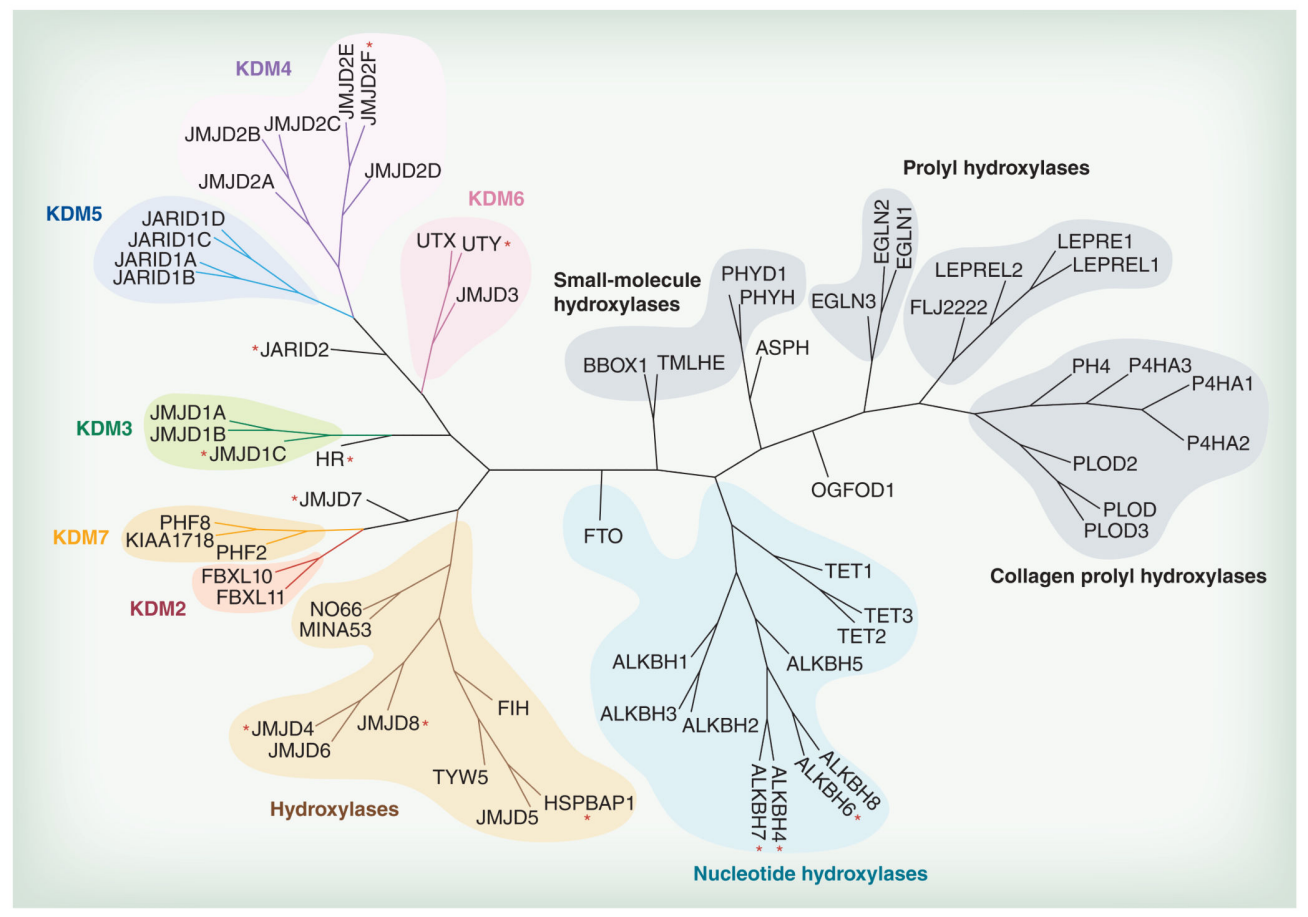
Phylogenetic tree of the human 2-oxoglutarate-dependent oxygenases Different subfamilies discussed in the text are highlighted in various colors. Red asterisks indicate members for which no enzymatic activity has been determined yet.

Cupins are ancient protein domains, found in archaea, bacteria and eukarya, and are often metalloenzymes with metal ion-containing active sites based around a histidine cluster – hence the jumonji C (JmjC) enzymes belong to a large family of metalloproteins that, despite low sequence similarities, share a common Fe^2+^- and 2-OG-dependent catalytic core. The human 2-OG oxygenase family consists of over 60 members, to which the different Jmj-type lysine demethylases contribute significantly (Figure 1). All members of this family share few conserved sequence motifs necessary for iron and cofactor binding (with exceptions discussed below), and are mechanistically defined by their ability to hydroxylate their specific substrates using an activated oxo-ferryl intermediate [6]. In brief, the catalytic sequence proceeds through distinct steps of 2-OG cofactor and molecular oxygen binding and activation, resulting in a highly reactive oxo-ferryl intermediate that reacts with the specific substrate atom, usually resulting in a hydroxylated substrate and concomitant CO_2_ and succinate formation. In terms of lysine demethylase reactions, the hydroxylated substrate is an unstable hemiaminal intermediate derived from the lysine methyl hydroxylation of the Nε -side-chain [6]. This intermediate then fragments into formaldehyde and a lysine side-chain decreased by a methyl group. Importantly, this mechanism allows to demethylate all possible Nε-methyl states (mono-, di- and tri-) found in methylated histone lysine residues and thus differs significantly from the mechanism of monoamine oxidases such as LSD1 and LSD2 (KDM 1A and B) [7] which are important mediators of chromatin function, and which can only demethylate mono- or di-methyl lysine states. Other important functions found in the human 2-OG family comprise hydroxylation of metabolic intermediates (e.g., phytanoic acid degradation [PHYH] [8], or involvement in carnitine synthesis [BBOX, TMLH] [9]), amino acids found, for example, in collagen (e.g., PLOD enzymes (Figure 1)) [10], or transcription factors (e.g., HIFα by 2-OG oxygenases such as FIH and PHD enzymes).

## Structural features of 2-OG enzymes

**Figure 2:**
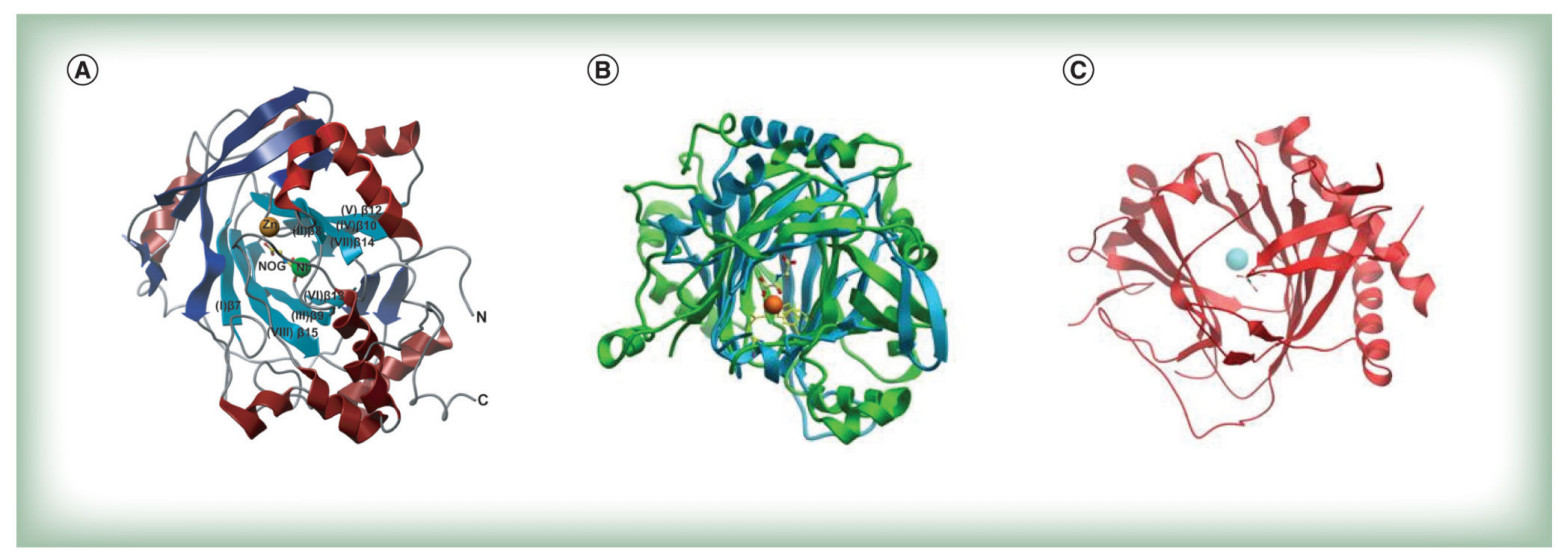
The overall fold of the catalytic JmjC domain in iron- and 2-oxoglutarate-dependent histone demethylases and nucleotide hydroxylases **(A)** Jmj prototype member JmjD2A (PDB ID: 2OQ7) in complex with Ni^2+^ (which replaces the endogenous Fe^2+^) and the 2-oxoglutarate competitive inhibitor *N*-oxalyl glycine (NOG). The double-stranded β-helical core elements are labeled I–VIII and colored cyan, the additional β-strands in blue and the helices in red. Ni^2+^ is shown as a green sphere and NOG as yellow sticks. **(B)** Overlay of the catalytic core (displayed are the active site metal, the Glu-His triad of active site residues and NOG) of human JmjD2A (green) compared to human ALKBH2 (PDB ID: 3BTX; light blue), indicating similar folding patterns of the catalytic domain. **(C)** Catalytic core of human methyladenosine demethylase FTO (PDB ID: 3LFM [14]) demonstrating the double-stranded β-helical fold and including the active site metal (blue sphere).

The JmjC domain is a double-stranded β-helical (DSBH) fold also called the jelly-roll fold or double Greek motif. The DSBH fold is composed of eight β-strands that form a β-sandwich structure comprised of two four-stranded antiparallel β-sheets (Figure 2) [11]. Most 2-OG oxygenases also contain additional secondary structure elements that surround the DSBH and define the different subfamilies. This includes additional β-strands that extend the DSBH, at least one helix on the C-terminal side of the DSBH, and inserts between the fourth and fifth β-strand. This DSBH core fold provides a rigid scaffold for the cosubstrates 2-OG and Fe^2+^, which are located in the more open end of the barrel (Figure 2). The iron is coordinated by two histidinyl residues together with a glutamyl or aspartyl residue in a conserved HxE/DxH motif found among 2-OG oxygenases. The 2-OG binding is less well conserved, and the cofactor coordinates the iron in a bidentate manner via its 2-oxo group and one of its 1-carboxylate oxygens, whereas the 5-carboxylate is usually bound to the side-chain of a basic residue (Arg/Lys) and to a hydroxyl group from a Ser/Thr or Tyr residue. The substrate binding varies considerably among the different subfamilies of 2-OG oxygenases/demethylases and the specific interactions identified thus far are beyond the scope of this review. However, crystallographic studies have revealed that it often involves residues from the first and second β-strand, together with strands and loops that extend the DSBH. Other protein domain modules, such as a plant homeodomain (PHD), Tudor, AT-rich interaction domain (ARID) or CxxC zinc finger motifs that vary between the different subfamilies, are usually also required for interaction with DNA/RNA or chromatin protein substrates [11-13]. An overview of the different protein domains as well as annotation of substrate specificities found in Jmj enzymes with defined or hypothetical roles in chromatin biology is given in Table 1.

## Jmj-enzymes as transcriptional regulators

Paradoxically, the founding member of the Jmj family, JARID2, lacks essential residues necessary for catalytic activity but nevertheless has been shown in various studies to be a key factor in mammalian development [3]. JARID2 shows sequence homology with the JARID1 (KDM 5) family which is defined by the presence of JmjC/JmjN and ARID domains. The molecular mechanism by which JARID2 modulates mammalian development is not well understood, however it is now established that it associates with Polycomb group (PcG) proteins in several cell types, including embryonic stem (ES) cells, and is also critical for ES cell differentiation [62-65]. PcG proteins were identified more than 30 years ago as regulators of homeobox (Hox) genes and development in *Drosophila melanogaster* [66,67] and also have a critical role in mammalian adult tissue homeostasis and cancer [68].

Importantly, these studies highlight the fact that most, if not all Jmj-type chromatin enzymes function as parts of transcriptional or chromatin protein complexes and in addition to their catalytic roles exert their function as scaffold proteins. The second major breakthrough in Jmj enzyme biochemistry and understanding as transcriptional regulators was therefore the discovery that members of the Jmj family indeed possess catalytic activity towards methyl-lysine histone substrates. This effort was spawned by the discovery of the first lysine demethylase, LSD1 [7,69] (which belongs to the amine oxidase family) followed by the characterization of distinct members of the Jmj family displaying methyl-lysine demethylase activity. These chronological discoveries led to the current and systematic nomenclature system of lysine demethylases (KDM) [70].

Identification of methyl-lysine demethylases was important, because post-translational modifications of N-terminal, unstructured tails of histone proteins had been previously recognized as key-components in the regulation and signaling of functional states of the epigenomic landscape. Accordingly, reversal of these modifications has important consequences in processes such as gene regulation or genome stability; for example, trimethylated lysine 9 of histone 3 (H3K9me3) indicates heterochromatic or repetitive regions, whereas H3K4me3 marks regulatory elements associated with active promoters or transcription start sites, and H3K27me3 often specifies developmentally repressed genes [71]. With the discovery of histone demethylation, lysine methylation of histone residues is now considered reversible and to respond dynamically to changes in metabolism or chromatin environment [72-74]. At present several classes of histone modifications and their respective enzymatic modification systems have been identified [72,75] and amongst their epigenetic substrate marks, lysine and arginine modifications are probably the best studied: acetylation and methylation of lysine residues, as well as methylation of arginine [72,75,76]. Whereas acetylation of histone tails is correlated with gene activation, the influence of histone methylation on regulating gene transcription depends on the exact residue methylated and the number of added methyl groups, both for arginine and lysine residues [72,75,76].

This review focuses on the description of Jmj-type chromatin hydroxylases and their involvement in human chromatin biology and associated pathologies. We will focus our description on the different KDM subfamilies, but also include the nucleotide hydroxylases as well as several further subfamily members with suspected nuclear or chromatin functions (Figure 1). For completeness, we will also briefly describe the monoamine oxidases of the KDM1 family despite being mechanistically distinct. It is worth noting that since the discovery of Jmj enzymes as mediators of histone demethylation [15] with its important consequences in chromatin biology and transcriptional regulation, not in every case have sufficiently stringent methods (e.g., product identification through mass spectrometry) been employed to properly characterize these enzymes. Therefore in several instances it remains to be established what their true endogenous substrates and inferred biological functions are.

## KDM1 subfamily

Starting with the groundbreaking discovery of LSD1 (KDM1A) as methyl-lysine demethylase [7], a series of studies revealed the enzymatic and biological roles of this amine oxidase that was able to demethylate histone methyl-lysine residues H3K4me2/1 and H3K9me2/1 in an FADH-dependent reaction (summarized in [73,77]). Following this work, the paralog LSD2 (KDM1B) with activity towards H3Kme2/1 was discovered [78,79]. Analyses of mice with targeted deletion of LSD1 or LSD2 revealed their essential roles in development [78,80], and other studies highlight the essential role of LSD1 in embryonic [81,82] and cancer stem cell biology [83,84]. Several investigations found that both KDM1 forms are aberrantly expressed in a variety of human tumor types (see [73]) and, taken together, provide the rationale for current inhibitor development. LSD1 is found in a variety of chromatin complexes, which include components such as HDAC/CoREST, BRAF35, BHC80 and noncoding RNA [85], and possibly the NuRD remodeling complex [86]; in these cases LSD1 functions as a repressor through its H3K4me2 demethylase activity. In contrast, LSD1 can associate with nuclear hormone receptors such as the androgen or estrogen receptor [69,87], and in these situations LSD1 shows H3K9me2 demethylase activity, thereby promoting target gene transcription. Taken together, the KDM1 enzymes are involved in various context-specific functions (e.g., in transcription initiation, enhancer modification or chromatin remodeling [73]). Interestingly, LSD1 is able to demethylate other nonhistone, chromatin-associated factors such as p53 or DNA methyltransferase 1 (DNMT1) [80,88], indicating that lysine demethylase substrates are not restricted to histone molecules.

## KDM2 subfamily

Soon after the breakthrough discovery of histone lysine demethylation mediated by LSD1, mechanistically different members of the Jmj family were shown to catalyze removal of methyl-lysine histone marks [15]. The first enzymes characterized within this family were human FBXL10 and FBXL11 (KDM2B and KDM2A, respectively), catalyzing the demethylation of H3K36me2/1 histone chromatin marks [15], an activity that orthologous forms display as well. The full-length proteins show a domain organization comprising JmjC, CxxC, PHD, Fbox and leucine-rich repeats (Table 1). Fbox-containing domains are involved in formation of cullin-Fbox protein-E3 ubiquitin ligase complexes, which suggests a link between KDM2, histone ubiquitination and proteasomal degradation. The CxxC zinc finger domains are modules that recognize unmethylated cytosine residues in a CpG dinucleotide context. Usually unmethylated, contiguous stretches of CpG elements are often found overlapping with a large fraction (up to 70%) of promoter and transcriptional start sites (so-called CpG islands [CGIs]), enforcing the notion that they contribute to gene regulation by providing a suitable environment for chromatin complexes [89]. Elegant work has provided evidence that FBXL10 and FBXL11 are recruited to the majority of mammalian unmethylated CGIs [90-93] through their CxxC-Zn finger domains. KDM2 enzymes thereby contribute to a specific chromatin environment surrounding CpG sites, independent of the transcriptional states of the particular gene. For example, FBXL11 is bound to 90% of annotated CpG sites in murine ES cells, creates a unique H3K36me2 depleted signature that distinguishes CpG/promoter structures from bulk chromatin, and possibly provides a permissive environment allowing subsequently chromatin elements such as regulatory and transcription factors to bind [90]. Interestingly, FBXL10 shows a largely overlapping occupancy of binding sites when compared to FBXL11 [91-93] and, in addition, defines a CGI subset through binding to Polycomb repressive complex 1 (PRC1), which mediates histone 2A (H2A K119) ubiquitylation through the RING1B component of this noncanonical PRC1 complex. This is in line with data about the activity of CGI1033, the *Drosophila* KDM2 ortholog that regulates PRC1-mediated H2A monoubiquitylation, independent of its demethylase function [94] and, collectively, provides novel insights how PRC1 is directed to its target genes. The localization of KDM2 enzymes to unmethylated DNA regions is consistent with mass spectrometry studies using nucleosome preparations, demonstrating enrichment at H3K9me3/unmethylated DNA nucleosomes [95]. Other chromatin functions of KDM2 forms may be related to heterochromatin formation and ribosomal gene transcription [16,96].

These studies give evidence for the essential role of KDM2 genes during development and in general for an emerging picture of chromatin architecture and function. The general targeting of KDM2 enzyme forms to CpG-containing promoter regions can explain many of the various phenotypes observed in several biological systems. For example, by being part of the Oct4/Sox2 regulatory axis, KDM2 enzymes can regulate reprogramming, pluripotency and stem cell properties [97-102], associate with corepressors such as BCL-6 corepressor (BCOR) [98,103], or control senescence and proliferation through repression of the Ink4b locus [99,104,105] and hence KDM2 involvement in oncogenic processes has been analyzed [106-108]. From these studies, it is apparent that KDM2 enzymes exert their critical importance through enzymatic (i.e., H3K36me demethylase activity) and nonenzymatic scaffolding functions (i.e., as being part of chromatin protein complexes). In addition, it has been shown that FBXL11 regulates the activity of the transcription factor NF-κB through direct demethylation of methylated Lys218 and Lys221 in NF-κB, a modification that is installed through the methyltransferase NSD1 [17].

## KDM3 subfamily

In humans the evolutionarily conserved KDM3 subfamily comprises four proteins, JmjD1A-C and the related *hairless* (HR) gene product. These proteins differ considerably in amino acid chain-length but have two regions in common that are both required for demethylase activity: a C_2_HC_4_-zinc finger-like domain followed by a C-terminal ~220-residue-long JmjC-domain that shares approximately 65% overall similarities [18,19]. Both full-length and truncated versions of JmjD1A and B specifically demethylate H3K9me2/me1 substrates while no demethylase activity has been described for human JmjD1C or HR yet. The demethylase activity of JmjD1A is inhibited by iron chelators and divalent metals (Co^2+^ and Ni^2+^) [109] that replace the ferrous iron at the catalytic site, as well as by NO that forms a nitrosyl-iron complex in the catalytic pocket [110]. JmjD1A-C enzymes are expressed in many cell lines or tissues and are primarily localized to the nucleus. Several phosphorylation sites have been reported for all members in the KDM3 subfamily that could be important for substrate recognition and signaling, but the impact of these modifications is unknown at present [19,111].

JmjD1A was identified by its interaction with the androgen receptor (AR) and acts as a coactivator of AR-mediated transcription [18]. The protein is also known as KDM3A, JHDM2 or testis-specific gene A (TSGA) since it is highly and dynamically expressed during spermatogenesis. Male knockout mice are infertile with small testes and a severe reduction in sperm count, which suggests that JmjD1A positively regulates the expression of genes involved in sperm chromatin condensation and maturation [112,113]. JmjD1A-knockout mice also exhibit an adult obesity phenotype, which implies an important role in transcriptional control of metabolic genes in muscle and adipose tissue [114,115]. The protein is upregulated in human cells exposed to hypoxia and its expression is regulated by HIF-1α that binds to a specific hypoxia responsive element in the JmjD1A promoter [116]. The hypoxia signaling pathway plays an important role in tumor progression, and JmjD1A has recently been associated with many types of cancers. Significantly elevated levels have been reported in several human cancer cells [117] including bladder, lung, hepatocellular [118], prostate [119], colorectal [120] and renal cell carcinoma [121], and treatment of cancer cell lines with siRNA targeting JmjD1A results in significant suppression of proliferation [118], tumor angiogenesis and macrophage infiltration into tumor tissues [117]. Although the exact role of JmjD1A in tumor progression remains to be elucidated it is considered a potential therapeutic target in the treatment of cancer.

JmjD1B also has the synonyms KDM3B/JHDM2B/5qNCA. In humans, the gene is located in the 5q31 chromosomal region, which is frequently deleted in myeloid leukemias [122] and in breast cancer [123]. JmjD1B is a suggested tumor suppressor [124] that regulates the expression of the leukemic oncogene *lmo2* [125] and it is also found to specifically interact with the transcriptional repressor suppressor of cancer cell invasion (SCAI) [19]. JmjD1C/JHDM2C was originally referred to as TRIP8 [126]. Two transcripts have been reported for this gene where the major transcript (variant 2) consists of a TRI8H1 domain with the zinc finger motif, a TRI8H2 domain with a thyroid hormone receptor β-binding region, and a C-terminal JmjC domain [127,128]. JmjD1C is similar to JmjD1A, an AR-interacting coactivator (see above) [129], that is suggested to perform a transcriptional regulatory function in testes development by regulating the expression of the steroidogenesis marker p450c17, via SF-1-mediated interactions [130]. JmjD1C is expressed in a variety of human tissues and its reduced expression in human breast cancer tumors compared to normal breast tissues suggests a putative role in tumor suppression [129]. No H3K9me2/1 demethylase activity has thus far been detected for the human protein [19], although all the conserved residues known to be important for enzymatic activity are conserved. A recent report that used both mutational analysis of single residues and domain swapping of both the JmjC- and the Zn-finger domain with JmjD1A/B revealed that both the sequence identity within the JmjC domain and in the sequence N-terminal of the JmjC domain are important for enzyme activity [19]. In other KDMs it has been shown that single amino acid substitutions are sufficient to completely abrogate enzymatic activity [15] and it is possible that thus far unidentified residues involved in substrate binding differ between the different KDM3 homologs. Further studies are needed to elucidate whether JmjD1C utilizes other substrates or cofactors, or if it has a predominant scaffolding function.

The HR protein plays a critical role in the maintenance of hair growth but the underlying mechanisms are still unclear. The protein is expressed in the skin but also the CNS [131,132] where it is suggested to function as a corepressor for multiple nuclear receptors [133-135] including the thyroid hormone receptor, the retinoic acid receptor-related orphan receptors and the vitamin D receptor [136]. HR also interacts with histone deacetylases, including HDACs 1, 3 and 5, and this interaction supposedly is responsible for the corepressor activity observed for HR [137]. Mutations in the HR protein (C622G, N970S, D1012N, V1136D) are associated with congenital alopecia or atrichia with papular lesions [131,138]. These pathogenic mutants occur primarily within the JmjC and the Zn-finger domain and appear to be essential for its co-repressor activity [136].

## KDM4 subfamily

The KDM4 subfamily of histone demethylases comprises four members, JmjD2A-D. The proteins are defined by an N-terminal JmjN and JmjC domain that in JmjD2A-C is followed by two tandem PHD and tudor domains (Table 1). These enzymes catalyze the demethylation of H3K9me3/me2 with a preference for the tri-methyl state, a histone mark associated with gene repression and found in heterochromatin. In addition, JmjD2A-C catalyze the demethylation of H3K36me3 albeit at a lower rate [20]. A less well-studied repressive histone mark, H1.4K27me, is also reported to be demethylated by all KDM4 members [22]. JmjD2A-C isoforms are also found to catalyze demethylation of trimethyl-lysine peptides of chromatin repressors WIZ, CDYL1, CSB and G9a proteins [21], indicating that KDM4 members can antagonize chromatin repressive marks and regulate gene transcription. A further two members of the human KDM4 subfamily (JmjD2E and JmjD2F) are known to exist but are currently considered to be pseudogenes [139] due to lack of intronic sequences in their genes. However, *in vitro* studies demonstrate that at least the catalytic domain of human JmjD2E is highly active and shows similar substrate specificities as JmjD2D [20].

The involvement of JmjD2A, B and C in cancer is now unequivocally established, highlighting the importance of these enzymes as potential targets for drug discovery (reviewed recently [140,141]). Many studies over the past 6 years have focused on the role of JmjD2 demethylases in progression of hormone responsive and hormone non-responsive cancers in both prostate and breast, underpinning the coregulation with nuclear hormone receptors. For example, Cloos *et al*. [142] showed that JmjD2A, B and C are increased in prostate cancer tissues followed by studies demonstrating KDM4 enzymes to be coactivators of the androgen receptor [143,144], in addition to roles in androgen independent proliferation of prostate cancer cells [145]. More recently [146,147] it was demonstrated by siRNA approaches that JmjD2B regulates AR transcriptional activity and controls stability of AR by prevention of proteosomal degradation of the receptor. JmjD2C (GASC1) has been shown to be amplified in triple-negative (estrogen, progesterone and HER2 receptor-negative) breast cancers [148]. Gene silencing studies demonstrate clear antiproliferative effects of JmjD2A knockdown in (estrogen receptor [ER]-negative breast cancer cells [149], and downregulation of JmjD2A in ER-positive breast cancer cells leads to reduced cell proliferation associated with decreased levels of cyclin D1 [150]. Yang *et al*. [151] showed that JmjD2B is highly expressed in ER-positive primary breast cancer and that JmjD2B is a target gene for ER itself, underlining the evidence for the importance of JmjD2B in the etiology of ER-positive breast cancers [152,153]. JmjD2B and JmjD2C are targets for the hypoxia inducible transcription factor HIF1α in cancer cell lines [154], and hence are of possible importance in regulating transcriptional responses in a hypoxic tumor environment. A role for the lipopolysaccharide (LPS)- and TNF-inducible enzyme JmjD2D in regulating H3K9me3 levels, and hence activity of upstream enhancer elements in a variety of cell types, was recently demonstrated [155].

Given the wealth of publications on the KDM4 family and cancer, relatively little has been published on their role in epigenetic control of other key processes such as embryonic development or normal tissue remodeling. Recent studies revealed a function for JmjD2A in specification of neural crest cells in the early mouse embryo [156]. Conditional gene ablation studies and transgenic studies in mice provide further insights into the function of KDM4 enzymes in development, for example, the generation of mice with a knockout or overexpression of JmjD2A in the heart shows a role for JmjD2A in cardiac hypertrophy in response to cardiac stresses [157]. More recent studies provide insights into how KDM4 enzymes regulate cell fate determination. *In vitro* and *in vivo* studies [158] have demonstrated a role for JmjD2B in commitment of mesenchymal stem cells (MSC) to the osteogenic lineage by removing the repressive H3K9me3 marks from osteogenic lineage-selective genes such as DLX5, thus favoring osteoblas-togenesis over adipogenesis. JmjD2B has also been shown to be a target gene for the transcription factor C/EBPβ, a transcription factor that is involved in preadipocyte proliferation and differentiation. In this study, a knockdown of JmjD2B inhibited mitotic cell expansion and terminal differentiation of the preadipocyte cell line, 3T3-L1 [159].

More recently, several studies demonstrated a role for the H3K9me3 demethylase JmjD2D (KDM4D) in oncology, inflammation and drug metabolism. In mice, JmjD2D assists the nuclear receptor constitutive androstane receptor (CAR), a key regulator of drug metabolizing enzymes in induction of a neonatal metabolic gene program persisting through adult life [160]. JmjD2D also interacts with the tumor suppressor p53 and controls proliferation [140,161], and activity of cell-type specific enhancer regions in immune cells through H3K9me3 demethylation [155].

## KDM5 subfamily

As with many of the JmjC lysine demethylases, the KDM5 family is composed of multidomain members. These are defined by the presence of both JmjC and JmjN domains but also a DNA-binding, ARID domain [162] and a C_5_HC_2_ zinc finger motif as well as methyllysine- or methyl-arginine-binding PHD domains involved in histone-substrate recognition (Table 1) [163]. There are four genes described within the human family, namely JARID1A (KDM5A/RBP2), JARID1B (KDM5B/PLU1), JARID1C (KDM5C/SMCX) and JARID1D (KDM5D/SMCY). All KDM5 members demethylate the H3K4me3 histone mark [23-27], a signature indicative of transcriptional activation, and hence KDM5 are considered transcriptional corepressors. One important function appears to be as members of chromatin complexes, for example, JARID1A is found associated to the PRC2 Polycomb complex, important in establishing repressive chromatin marks during development, and JARID1C can be identified in complexes with other repressive chromatin modulators such as HDAC1/2/REST, or the H3K9 methyltransferase G9a [77,164]. KDM5 enzymes are found around the transcriptional start sites of a large set of target genes, where they are thought to fine-tune the transcriptional output, as indicated by modest transcriptional responses upon depletion of, for example, JARID1B – effects, which nevertheless are critical for cellular responses in development and disease [73].

JARID1A was discovered as an interaction partner of the retinoblastoma protein (Rb), a key regulator of cell cycle control and differentiation [165], hence its alias as Rb-binding protein (RBP2). Interactions between the two proteins can promote cellular differentiation [166], in addition to a role for JARID1A and demethylation of H3K4 in the control of cellular senescence [167]. JARID1A regulates HOX gene activity during development [23], underlining the importance of KDM5 enzymes in stem cell biology. The role of JARID1A also extends to other areas of cellular control, with an example being modification of the circadian clock period [168]. In cancer, the NUP98/JARID1A gene fusion has been described as a cryptic translocation involved in pediatric acute megakaryoblastic leukemia [169], and additionally the JARID1A locus was identified in a recent GWAS analysis as a susceptibility gene in ankylosing spondylitis [170]. Importantly, JARID1A has been implicated in facilitating an altered chromatin state that promotes drug-tolerant subpopulations of cancer cells [171].

JARID1B displays an important yet complex role in stem cell biology by blocking differentiation in embryonic and hematopoietic stem cells [172,173], however in a different study it was found to be dispensable for embryonic stem cell maintenance and critical for neural differentiation [174]. JARID1B is also implicated in the control of cellular senescence [167]. It is a deregulated factor in several cancer types, for example, it can suppress tumor progression in breast cancer cells [175], and is specifically expressed in melanoma [176], as well as breast cancer [177]. The involvement of JARID1B in cancer stem cell maintenance highlights its role in tumorigenesis [178,179].

JARID1C and JARID1D are located on the X- and Y-chromosomes respectively and may well have diverged from a single ancestral sex chromosome [180]. Intriguingly, this segregation to the gonosomes offers some interesting prospects regarding sex-specific gene regulation, however, it should be noted that JARID1C is not subject to X-inactivation [181]. The ubiquitously transcribed JARID1C is known for its role in X-linked intellectual disability (XLID). It is highly expressed in brain tissue and the gene is found to be frequently mutated in this condition of male cognitive impairment [180]. There is also a potential role for JARID1C variants in autism spectrum disorder [182]. Taken together, these data present evidence for an important role for JARID1C in normal brain function, by regulating expression of important neuronal genes [164]. In tumor biology, JARID1C interacts with the von-Hippel–Lindau protein and has been shown to regulate gene expression and to suppress tumor growth [183].

JARID1D transcripts are detected in a wide range of male tissues and a JARID1D-derived peptide has been identified as the male-specific histocompatibility antigen (H-Y) [184-186]. The JARID1D genomic locus is associated with a region of the Y-chromosome designated azoospermia factor region; a region involved in spermatogenesis. It is thought that JARID1D interacts with the meiosis-regulatory protein MSH5 during this process [187]. Furthermore, deletion of this gene has been detected in prostate cancer [188]. It has been shown that PCGF6/MBLR can directly interact with JARID1D to enhance its demethylase activity via a C-terminal domain [26].

## KDM6 subfamily

Since the identification of JmjD3 and UTX as H3K27 demethylases in 2007 [28-32], numerous publications have emerged describing the functions of H3K27 demethylase enzymes in various biological contexts such as development, inflammation and oncology. The human KDM6 subfamily consists of three distinct members, namely JmjD3, UTX and UTY with documented histone lysine demethylase activities for UTX and JmjD3 (KDM6A and B). Whereas UTX and UTY are located on X and Y chromosomes, respectively, JmjD3 is found on chromosome 17. All KDM6 members have a JmjC domain followed by a GATA-like DNA-binding domain, whereas UTX and UTY also have N-terminal located protein interaction (tetratricopeptide repeat [TPR]) domains (Table 1) [189]. Whereas the catalytic role of JmjD3 and UTX is undisputed, critical scaffolding and protein interaction functions of all KDM6 members have been recognized. For example, Sola *et al*. reported that during mouse neural stem cell differentiation, the N-terminal region of JmjD3 stabilizes the key transcription factor p53 in a demethylase-independent manner and contributes to nuclear localization of p53 [190], or during T-cell development JmjD3 associates with Tbox proteins independent of its catalytic role [191,192]. However, a key mechanism of JmjD3 and UTX appears to be their ability to remove H3K27me3 chromatin marks which anchor Polycomb repressive complexes and are often found in ‘bivalent’ chromatin domains thought to be in a transcriptionally ‘poised’ state [193].

JmjD3 is expressed in human, rhesus monkey and mouse oocytes during metaphase [194], and plays a role in oocyte preimplantation (e.g., in bovine fertilization), since knockdown of JmjD3 in oocytes inhibited H3K27me3 demethylation and impacted blastocyst [195], and later endoderm and mesoderm development. This is accomplished through methylation of bivalent chromatin domains and their poised transcriptional states [71], by removal of H3K27me3 marks, thus enabling expression of key developmental genes such as WNT3, DKK1 or FOXH1 involved in Wnt and Nodal pathways [196,197]. Multiple studies highlight the key role of H3K27 demethylases in cellular differentiation, for example, in later stages of embryo development, JmjD3 is largely involved in regulation of genes responsible for body and limb morphogenesis, including HOX and T-box transcription factors [198,199]. However, here the enzymatic function of JmjD3 appears to be at least in part dispensable. The importance of H3K27 demethylases is further underpinned by their roles in induced pluripotent stem cell (iPS) biology. Silencing of JmjD3 in mouse embryonic fibroblasts (MEFs) during iPS reprogramming enhanced iPS formation, whereas ectopic expression of JmjD3 inhibited reprogramming, an effect that is mediated through both demethylase-dependent and -independent pathways [200]. Where as the demethylase-dependent function mediates removal of H3K27me3 repressive marks on genes involved in reprogramming such as Ink4/Arf, the demethylase-independent pathway involves PHF20 for ubiquitination, thereby leading to degradation [200]. Other work has shown that JmjD3 is involved in bone cell differentiation, for example, knockdown of JmjD3 caused significantly reduced osteogenic differentiation of mesenchymal stem cells to osteoblasts [158]. Osteoclast development is partly regulated by H3K27 demethylation, by controlling H3K27me3 levels at the promoter of the key osteoclastogenic transcription factor NFATc1 [201]. A number of studies indicated that JmjD3 also plays an important role in neural differentiation and commitment [202,203]. Here, JmjD3 is one of the early neural differentiation regulators modulated by BMP4 in neural stem cells and is crucial to activate TGFβ-responsive genes [204,205]. In retinoic acid (RA)-induced neural differentiation, an increased expression level of JmjD3 was shown to remove H3K27me3 at the HOX gene regulatory elements [199]. Mash1, a key gene in RA neuronal differentiation, is also regulated by a similar mechanism [206], whereas silencing of an RNA binding protein induces expression levels of JmjD3 in RA neurogenesis [207]. Importantly, a JmjD3-knockout in mouse resulted in perinatal lethality, affecting the pre-Botzinger complex (PBC) complex, which is the pace-maker of the CNS respiratory rhythm generator [202]. The critical role of UTX in developmental effects is underscored by the discovery of mutations in the human gene associated with Kabuki syndrome, an intellectual disability syndrome also associated with mutations in the H3K4 MLL methyltransferase [208,209].

A role in inflammation was found for KDM6 enzymes, since JmjD3 expression in macrophages is regulated through the NF-κB signalling pathway in LPS-stimulated macrophages [28]. It was found that JmjD3 associates with 70% of LPS-inducible genes [210,211], though it is unclear whether JmjD3 is directly involved in transcription of these genes [212]. A direct effect on regulating TNF promoter K27 methylation and transcription was demonstrated in a study using a H3K27 demethylase inhibitor [189]. Stimulation of mouse macrophages by IL-4 induces JmjD3 via the STAT6 pathway and leads to activation of several anti-inflammatory genes [213]. In addition, in mice, the polarization of anti-inflammatory macrophages during helminth infection is modulated by JmjD3 [214]. In T cells, JmjD3 is involved in T-helper cell lineage commitment [191], through interaction with T-box transcription factors. Expression of HPK1/MAP4K1, a kinase that negatively regulates T-cell immune response, is influenced by JmjD3-mediated demethylation of H3K27 [215]. Overexpression of JmjD3 causes upregulation of HPK1, which in turn suppresses T-cell responses. Hence, blocking of JmjD3 activity could be a potential therapeutic approach for the treatment of systemic lupus erythematosus [215]. During wound healing, JmjD3 and UTX are upregulated at the wound site and control epidermal differentiation [216,217]. JmjD3 is also overexpressed in vasculitis [218] where it regulates expression of Itaga2b and Mpl in megakaryocytes [219].

Unsurprisingly, the critical function of H3K27 demethylases (KDM6 enzymes) in controlling key developmental factors is reflected also through their involvement in oncology. Importantly, the particular role (either as tumor suppressor or oncogene) depends on the particular cellular context. Numerous studies have demonstrated overexpression of JmjD3 in cancer cell lines or tumor biopsies, also associated with increased proliferation or control of anti-apoptotic genes such as Bcl-2 [211,220-223], or for example aberrant repressive PcG methylation patterns in medulloblastoma [224,225].

However, in several instances KDM6 enzymes can also be regarded as tumor suppressor genes by counteracting the oncogenic function of PcG proteins, for example by controlling the INK4A/ARF locus that comprises tumor suppressor genes p16INK4A and p14ARF involved in cellular senescence. For example, in cells undergoing oncogenic stress, JmjD3 is induced by RAS/RAF signaling pathways and activates P16/INK4a and p14/ARF, causing p53-dependent cell cycle arrest [226-228].

JmjD3 is involved in the epithelial–mesenchymal transition (EMT), and is overexpressed in invasive breast carcinoma. Knockdown of JmjD3 prevented breast cancer infiltration, and overexpression of JmjD3 affected the expression of a range of adhesion molecules, including downregulation of E-cadherin and upregulation of N-cadherin and fibronectin. Detailed analysis revealed that during TGFβ stimulated EMT, JmjD3 removes H3K27me3 at the promoter of an EMT-inducer gene, SNAI1, thus allowing transcription of the gene [229]. In SW480-ADH colon cancer cells, calcitrol (vitamin D3, 1,25-(OH)2D3 induces JmjD3 expression thus controlling critical target genes. Knockdown of JmjD3 induced SNAI1-mediated EMT, upregulation of mesenchymal markers and downregulation of epithelial proteins [230,231]. Expression of JmjD3 correlates with expression of vitamin D receptor but is inversely correlated with expression of SNAI1 in 96 human colon tumors [230,231]. Lastly, mutations found in the UTX or JmjD3 genes suggest a possible role in the oncogenic process [232,233].

Little is currently known about the function of UTY, a gene showing a large number of splice variants [234], but encodes a minor HLA-B8 HY-antigen, implicated in graft/host interactions [235]. A recent genetic study identified downregulation of UTY in the predisposing haplogroup I of the Y chromosome in coronary artery disease [236].

## KDM7 subfamily

The human KDM7 subfamily consists of three members: KIAA1718 (KDM7A), PHF8 (KDM7B) and PHF2 (KDM7C), showing a characteristic domain organization with an N-terminal PHD domain, and a catalytic Jmj domain (Figure 1 & Table 1). The C-termini are comprised of coiled–coiled domains and structurally undetermined amino acid stretches. These C-terminal regions have been shown to be important for association with RNA polymerase or transcription factors such as the retinoic acid receptor (RAR) and possibly cell cycle regulators such as E2F1, and contain nuclear localization signals or phosphorylation sites [237]. Several reports unequivocally demonstrate a catalytic role of KDM7 members in demethylation of repressive histone marks such as H3K9me1/2, H3K27me1/2 or H4K20me1 (summarized in [237]) with distinct differences in substrate specificities among the various enzymes. The critical importance of these activities in mediating transcriptional coactivation is highlighted through association with H3K4me3 marks (through the conserved PHD modules [34]), and oxidative removal of repressive histone marks. This creates a permissive chromatin environment by facilitating transcription through subsequent acetylation of lysine residues after methyl mark removal which is accomplished through KDM7 enzymes [237]. The cooperative role of histone marks in KDM7 mediated coactivation is further emphasized through phosphorylation of Ser 3 of histone 3 (H3S3), an important histone mark during mitotic progression, which also prevents KDM7 binding to mitotic chromosomes [238,239], in addition to the cell cycle-dependent phosphorylation of, for example, PHF8, a critical event in cell-cycle progression [240]. The importance of KDM7 members in gene transcription is demonstrated through chromatin immunoprecipitation followed by next-generation sequencing, showing promoter occupancy at a large fraction of transcriptionally active genes [33,239-241]. Besides their roles in RNA polymerase II (RNA Pol II)-mediated transcription, at least PHF8 and PHF2 are also involved in RNA Pol I-mediated RNA gene transcription [33,35,242]. The pathophysiological roles of KDM7 members include involvement in cancer, such as RARα coactivation in pro-myelocytic leukemia [243], regulation of proinflammatory responses [244] and development (e.g., of neural tissues) [245]. PHF2, a H3K9me2 demethylase [33], has been shown to play a role in breast cancer [246]. Several mutations described in PHF8 are causative of Hamel–Siderius syndrome [247-249], an X-linked mental retardation often accompanied by developmental malformation such as cleft lip/cleft palate [250] or microdeletions in autism disorders [251], in line with observations on the critical roles of KDM7 enzymes during neuronal development [36]. Interestingly, another 2-OG enzyme, trimethyllysine hydroxylase found within the small molecule branch (TMLHE, Figure 1), was identified along with PHF8 in an X-chromosome exome sequencing study of autism and intellectual disability cases [252].

## Other putative Jmj-type demethylases & amino acid hydroxylases

This group (see Figure 1) of human 2-OG oxygenases comprises a functionally diverse group of enzymes several of which have well-described roles, such as factor inhibiting HIF (FIH), or which have been rarely studied to date (e.g., JmjD8). These enzymes have, beside the Jmj core domains, few predicted or structurally verified additional domains, the lack of chromatin-binding domains especially suggests potential functions other than histone modification. Indeed, with few studies conducted thus far, the role in histone modification appears, in most instances, secondary at best. Nevertheless, for several of these 2-OG enzymes, hydroxylation of amino acids (e.g., asparagine or histidine residues) in nonhistone proteins has been clearly shown by using mass spectrometry techniques.

Among the best-studied members of the Jmj family is FIH, an asparaginyl hydroxylase that modifies an asparagine residue (Asp803) in the C-terminal transactivation domain of HIFα proteins. These transcription factors regulate key programs involved in metabolic adaptation to low O_2_ tension, such as energy metabolism, redox and pH homeostasis, and O_2_ supply, as well as many other functions [253]. FIH-mediated HIFα hydroxylation is important to regulate interaction with the transcriptional coactivator p300, a function that is impaired under low oxygen levels [254]. HIFα activity is regulated by FIH in concert with prolyl hydroxylases (see Figure 1) such as PHD1 and PHD2, which regulate proteasomal degradation of HIFα under normoxic conditions. In normoxia, PHD and FIH enzymes act synergistically to degrade and inactivate HIF-1α. Under hypoxic conditions, the PHD enzymes no longer hydroxylate HIFα, leading to stabilization and accumulation of the HIF-1α subunit. FIH remains active at this stage and continues to repress HIFα activity until conditions of severe hypoxia occur, where FIH ceases to hydroxylate the asparagine residue in the C-terminal domain and releases HIFα repression. FIH-null mice do not display a phenotype related to increased HIF or hypoxia responses, but show significant metabolic changes such as reduced body weight, elevated metabolic rate and resistance to effects of high-fat diet [255,256]. FIH is important in several cancer types, for example, FIH expression is regulated by miRNA-31 in head and neck squamous cell carcinoma [257]. Low nuclear expression of FIH is a prognostic factor for poor overall survival in clear cell renal cell carcinoma [258]. Inhibition of FIH-1 in cell cultures of cell renal cell carcinoma decreases cells expansion and increases apoptosis by regulating HIFα [259]. Over expression of FIH and PHD HIF hydroxylases in non-small-cell lung cancer is indicative of a poor prognosis [260]. FIH is also expressed in invasive breast carcinomas [261], and is found to be inhibited by gankyrin, an oncoprotein expressed in hepatocellular carcinomas, causing an increased HIF and VEGF expression resulting in hemangioma [262].

More recently, it has been demonstrated that FIH can also modify asparagine, aspartate and histidine residues in ankyrin repeat proteins such as NF-κB or Notch [48-50] indicating hydroxylation of amino acid residues as a widespread function that awaits further investigations.

MINA53 is closely linked to expression of the transcription factor c-Myc [263], a gene associated with cell growth, proliferation, loss of differentiation and apoptosis [264]. MINA53 has been reported to demethylate H3K9me3 [265], but this activity is controversial since a lack of demethylase activity has been reported by several groups. More recently it was shown that MINA53 catalyses histidyl hydroxylation of ribosomal protein L27a, suggesting a function in ribosomal assembly and function [39]. A series of papers have confirmed MINA53 effects on cell differentiation, and overexpression of MINA53 in various cancers appears to be linked to oncogenesis [266-273]. The role of MINA53 has been especially well characterized in lung cancer, making it a potential prognostic biomarker for the disease [270,273]. MINA53 expression was also found to be induced by silica particles [273], and the protein plays a key regulatory role in immunity, for example, in allergen-induced inflammatory response [274] and, importantly, in differentiation of proinflammatory TH17 cells [275]. Taken together these studies conclude that MINA53 is an important regulator in inflammation and oncology, however the mechanistic link between its enzymatic activity and those cellular phenotypes awaits clarification.

The closely related oxygenase NO66 (also known as MAPJD) was first identified and characterized in 2004 by Eilbracht *et al*. in purified nucleoli from *Xenopus laevis* oocytes [276]. NO66 shows significant sequence homology to MINA53 (34% sequence identity) and is reported to demethylate H3K4 and H3K36 [40], and similar to MINA53, to catalyse ribosomal histidinyl hydroxylation, in this case on ribosomal protein L8 [39]. During mouse embryonic stem cell differentiation NO66 is recruited by PHF19, a component of the Polycomb repressive complex PRC2, to stem cell genes resulting in loss of H3K36me3, transcriptional silencing and PRC2-dependent methylation of H3K27 [277]. NO66 regulates osteoblast differentiation and bone formation by inhibiting Osterix (Osx) [40], a key transcription factor essential at later stages of bone development, accordingly Osx-null mice have a normal cartilage development, but lack bone formation. Conversely, knockdown of NO66 in differentiating osteoblasts derived from mesenchymal stem cells causes accelerated differentiation and mineralization of osteoblasts [40]. The interaction between NO66 and Osx was recently mapped to a conserved hinge domain, that links the N-terminal JmjC domain and C-terminal wHTH domain of NO66 (Table 1) [278]. The protein is overexpressed in non-small-cell lung cancer, and postulated to be a potential drug target [279].

JmjD4 is a poorly characterized oxygenase whose enzymatic activity has not been characterized, and shows approximately 30% sequence similarity to JmjD6. JmjD5 (also classified as KDM8) plays a critical role in the regulation of cell cycle by inhibition of HDAC recruitment and by activating the cyclin A1 locus, postulated through demethylation of histone H3K36me2 [42], although this activity needs further investigation. Instead, a hydroxylase activity of JmjD5 appears to control the half-life of transcriptional regulators such as NFATc1 through promotion of E3-ligase association and proteasomal degradation of the transcription factor [43]. JmjD5 deficiency results in a short-period circadian phenotype both in mammalian cell cultures and *Arabidopsis* plants [280]. JmjD5 is widely overexpressed in several types of tumors (e.g., in leukemia and breast cancer [281]) and a knockout in a MCF7 breast cancer cell line resulted in reduced proliferation [42], and its function appears to be interrelated with the tumor suppressor p53. Ablation of murine JmjD5 causes severe growth retardation and ends in embryonic lethality [281,282].

JmjD6, initially characterized as phosphatidyl serine receptor [283], a function that together with its postulated methyl arginine demethylase activity [44] has not yet been confirmed, catalyzes the hydroxylation on the carbon side-chain of lysyl residues located in arginine–serine rich regions of, for example, splicing factors [45,284] or might modify single-stranded RNA [285], indicating involvement in RNA modification and function. In mice, the gene is indispensable for normal development of many tissues such as brain, eyes, lung, kidney, liver and intestine at different stages of embryogenesis [286-288]. Furthermore, JmjD6 plays an important role during heart development since ablation of its function is associated with complex cardiopulmonary malformations that resemble the human congenital heart syndrome tetralogy of Fallot [289]. Overexpression of JmjD6 protein has now been strongly linked to poor prognosis in breast cancer [290] and in lung adenocarcinoma [92]. JmjD7 is a poorly characterized enzyme located on chromosome 15; a fusion with phospholipase represents a novel isoform of this lipid metabolizing enzyme [291]. JmjD8 is linked to cell proliferation and cancer but its enzymatic and cellular functions remain unknown. JmjD8 siRNA knockdown in SCC23/MET cells, a cellular model for squamous cell carcinoma, inhibited invasion of the cells by 80%, suggesting an inhibitory effect on cell proliferation [292]. The rat ortholog of human HSPBAP1 (named PASS1) was identified in 2000 by Liu *et al*., associated with the heat shock protein hsp27 [293]. PASS1 is expressed in various tissues, but shows high expression in the testis and kidney, whereas human HSPBAP1 is abundant in the thymus and pancreas, as indicated by reverse transcription-PCR analysis [294]. HSPBAP1 inhibits the function of hsp27, which has been shown to be neuroprotective in animal models of motor neuron disease and peripheral nerve injury [295,296]. HSPBAP1 is expressed in neuronal and glial cells in the temporal lobe of patients with IE, with no brain expression in control tissues [296]. A follow-up study tested samples from the temporal neocortex taken from intractable epilepsy patients showing significant expression of HSPBAP gene over the controls [297], however the enzymatic role of HSPBAP1 remains unclear. TYW5 acts as a tRNA hydroxylase and at present a biological function has not been reported [52].

## Nucleotide hydroxylases

The distantly related group of nucleotide hydroxylases comprises, in humans, the ALKBH members (ALKBH 1–8) and the TET 1–3 and FTO enzymes (see Figure 1). Despite low sequence identities they share the conserved sequence and structural elements found within the 2-OG oxygenase family (Figure 2). ALKBH enzymes were initially identified as mammalian orthologs of the *E. coli* AlkB DNA repair enzyme [53], important in the adaptive repair response to alkylated DNA residues such as 1-methyladenosine and 3-methylcytosine in single-stranded DNA [53,54]. However, more recent investigations have demonstrated that their physiological role in humans extends well beyond DNA damage repair (see below).

ALKBH 1–3 have been studied extensively and their role in DNA and RNA damage repair has been established [54,298-303]. Although they display distinct but overlapping subcellular distribution and substrate specificities, they are critically involved in various types of cancer and indicative of cancer drug responses [304-311]. Human ALKBH4 supposedly interacts with chromatin-binding proteins such as the histone acetyltransferase p300 [312], however, no enzymatic function with nucleotide substrates has been demonstrated yet. Recently, it was found that demethylation of a monomethylated lysine residue in actin by ALKBH4 regulates actin-myosin interactions, of importance in cytokinesis and cell migration [55].

The landmark discoveries of two Jmj members as RNA demethylases have opened new avenues in under-standing the relationships between RNA metabolism, gene regulation and human physiology and disease [313,314]. Initially found in FTO [315] and later extended to ALKBH5 [56], demethylation of N^6^-methyladenosine (m^6^A) residues found in several types of RNA species was identified, with a reaction sequence analogous to Nε demethylation of lysine residues [313]. The discovery of reversible methylation of m^6^A, a prevalent methyl modification found in mRNA and noncoding RNA, the effects of chemical inhibition of RNA methylation on transcription, processing, translation and the analysis of animal or clinical phenotypes therefore suggest a fundamental role of m^6^A demethylation in human physiology [313,314]. The critical role of these enzymes was already suggested by the identification of FTO variants as risk alleles for BMI and obesity [316]. Subsequently, the *in vitro* activity as demethylase for 3-methylthymine and 3-methyluracil found in single-stranded DNA and RNA was established, indicating the distant relationship with the ALKBH-type nucleotide hydroxylases [60,61]. Although the subject of *in vivo* activity is still a matter of debate, data indicate that m^6^A is a physiological substrate of FTO, as evidenced from knockdown studies [315]. The role of FTO in maintenance and regulation of energy homeostasis and food intake has been demonstrated in animal models [317] and through GWAS relationships to insulin resistance and obesity [318,319], Alzheimer’s disease [320], cardiovascular disease [321] and renal failure [322], as well as breast and colorectal cancer [323,324] have been established. A loss-of-function homozygous mutation in the FTO gene shows a defect in normal brain and cardiac development [325], in line with previous observations in animal studies.

Other currently uncharacterized members of the ALKBH subfamily comprise members ALKBH6 and 7. Human ALKBH8 has been recently identified as a methyltransferase acting upon 5-carboxymethyl-uridine and catalyzes an important step in tRNA synthesis, is involved in survival after DNA damage [57], and may contribute to cancer progression [326].

The subgroup of TET enzymes (Figure 1) was named after the ten-eleven translocation (t10;11)(q22;q23) found in rare cases of acute myeloid and lymphocytic leukemias [327,328]. The translocation fuses the mixed-lineage leukemia (MLL) gene on chromosome 10 with the TET1 gene on chromosome 11. Subsequently the relationship to the 2-OG family and the activity as hydroxylases of 5-methylcytosine with the consecutive formation of 5-hydroxymethylcytosine, 5-formylcytosine and 5-carboxycytosine was established in a series of elegant studies [58,59]. Identification of TET enzymes as 2-OG members was a challenging task, due to the large insertions found with the catalytic domains (Table 1) [58], that made correct assignment challenging. Oxidized methylcytosine marks are found at promoters, enhancers and gene bodies, depending on the tissue examined. The chemical modification sequence of oxidation of methylcytosine forms the basis for an enzymatic mechanism to remove the DNA methylcytosine modification, shown to be critical for epigenetic regulation of gene transcription. Several mechanisms of methylcytosine demethylation might apply, including passive demethylation during cell division, or active enzymatic mechanisms acting on the oxidized intermediates. The TET-modified cytosine residues can be actively removed through a base-excision mechanism using DNA glycosylases, or might involve activation-induced cytidine deaminase (AID) and APOBEC constituting a deamination reaction of 5-hydroxymethylcytosine to 5-hydroxyuracil, followed by glycosylase and cytosine replacement. Other mechanisms might involve enzymatic decarboxylation by a yet unidentified decarboxylase, or dehydroxy-methylation by DNA methyltransferases [329]. The importance of the TET enzymes in stem cell biology and development (summarized in [329]) is at present not completely understood, however, especially in myeloproliferative types of cancers, their critical roles have been now established [327,328,330].

## Conclusion & future perspective

Over the last decade we have witnessed significant progress in understanding the function that many members of the Jmj family have in human physiology and disease. This advancement went hand-in-hand with technological improvements in, for example, allowing interrogation of epigenomic changes at single base-pair resolution, providing unprecedented details and mechanisms how chromatin marks sculpt and regulate cellular phenotypes and features such as proliferation, differentiation, metabolism and genomic stability, among others. Undoubtedly, the discovery of hydroxylation as a mechanism to revert *N*-methylation found in nucleic acids or proteins is a milestone that has triggered substantial progress in identifying the role of this post-translational modification in chromatin biology, and defining epigenomic elements in genome biology in general.

We have here attempted to summarize the current knowledge of human Jmj enzymes with particular emphasis on histone lysine modification and their roles in human disease. It is apparent from this body of research literature that the involvement of Nε-methyl lysine demethylases in disease is partially related to putative functions of the epigenomic elements they modify, however, recent data suggest that several histone demethylases bind to a large number of genes, but display only modest effects on target gene expression when depleted, suggesting a ‘fine-tuning’ effect of these enzymes [73]. Moreover, they are often found bound to chromatin without their substrate mark present, suggesting a ‘guardian’ function to ‘protect’ chromatin against aberrant modifications [73]. This indicates that the KDM-Jmj hydroxylases are not necessarily involved as on-off switches (as found, for example, in the kinase-phosphatase paradigm of signal transduction), but support other roles, highlighted by the fact that a scaffolding function is of critical importance in various settings, (e.g., as observed for JARID2 or the KDM6 enzymes). However, a substantial fraction of the Jmj enzymes (Figure 1) remains enigmatic in terms of substrate specificity or enzymatic activity, calling for further systematic screening activities, including non-histone substrates. In fact, the discovery of Jmj-mediated oxidation of nucleic acids or nonhistone proteins suggests that the substrate spectrum is much larger than anticipated. Furthermore, there might have been substantial misleads in identifying substrate specificities by employing inappropriate assays, so it appears mandatory to stringently evaluate those biochemical parameters to deduce cellular functions. Laudable efforts to map the cellular ‘lysine-methylome’ [332] have recently demonstrated that nuclear lysine methylation constitutes approximately 40% of cellular Nε-lysine methylation, including transcription factors and chromatin modifiers besides histone proteins, thus providing a wealth of testable novel substrate hypotheses for Jmj enzymes.

An important aspect deserving further research efforts is to understand the regulation of Jmj enzymes themselves. Intriguingly, some of the KDM enzymes are inducible by extracellular stimuli (e.g., LPS or inflammatory cytokines and growth factors) exemplified by JmjD3 or JmjD2D, and post-translational modifications or domain arrangements of the enzymes have an impact on enzyme activity as seen with PHF2, indicating that substrate analyses need evaluation in the context of cellular expression and specific growth conditions. It is also becoming increasingly clear, that regulation of Jmj activity through levels (normoxic vs hypoxic) of the cosubstrate O_2_, or indirectly through HIFα-mediated responses, reactive oxygen species or metabolic intermediates is of importance [154,253,333,334]. Taken together, these research lines will undoubtedly reveal novel facets in regulation of the epigenetic landscape and its impact on human disease. However, to evaluate and harness the potential of Jmj-enzymes as potential drug targets, chemical biology efforts to provide tools for better understanding of biology are mandatory [335]; fortunately recent efforts from several laboratories reveal that this class of enzymes indeed is chemically tractable, expanding the chemical toolbox of epigenetic inhibitors and possibly allowing to modulate disease outcome by targeting Jmj-type oxygenases.

## Figures and Tables

**Table 1:** Domain organization and substrates of human Jmj-type oxygenases

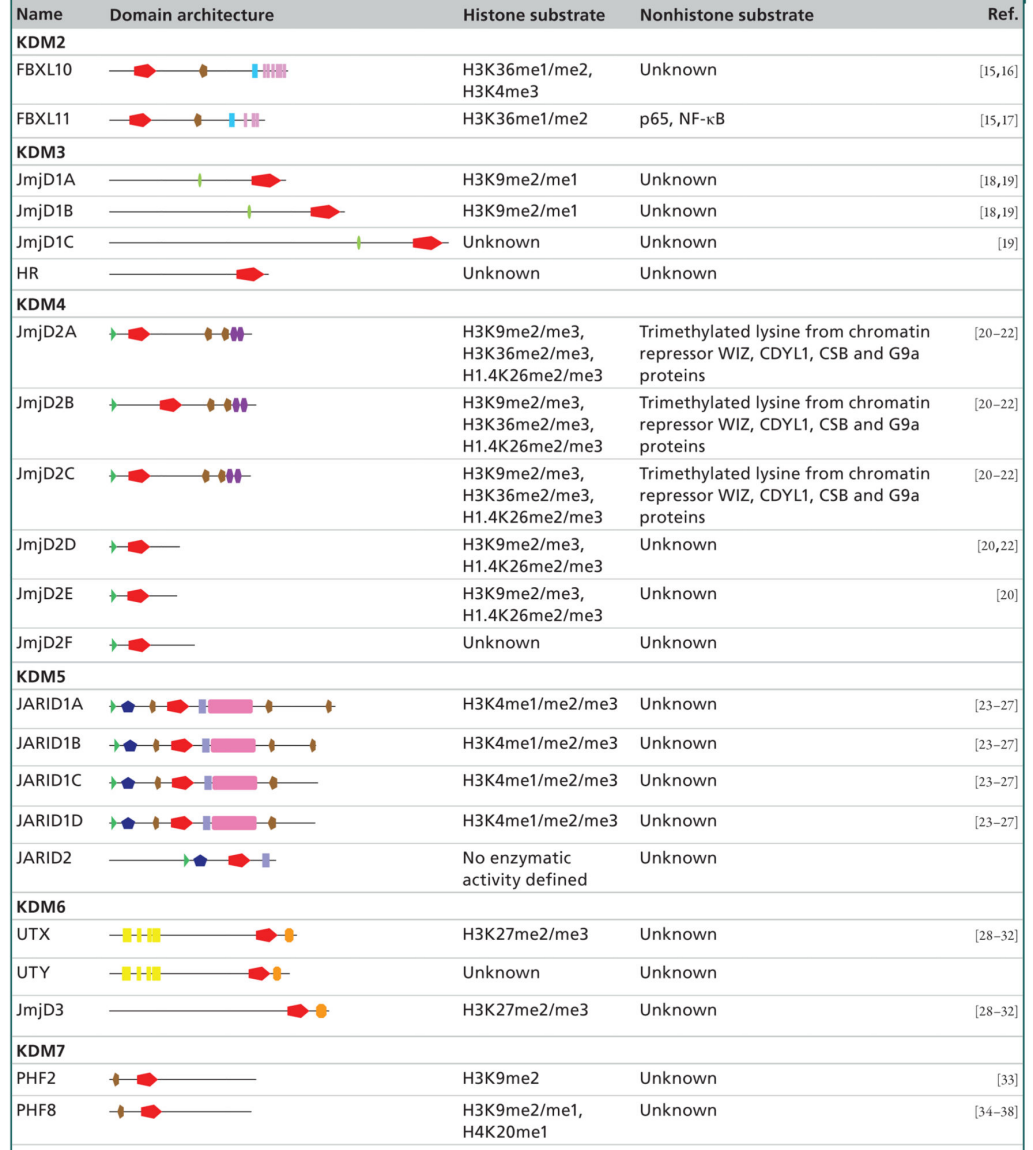
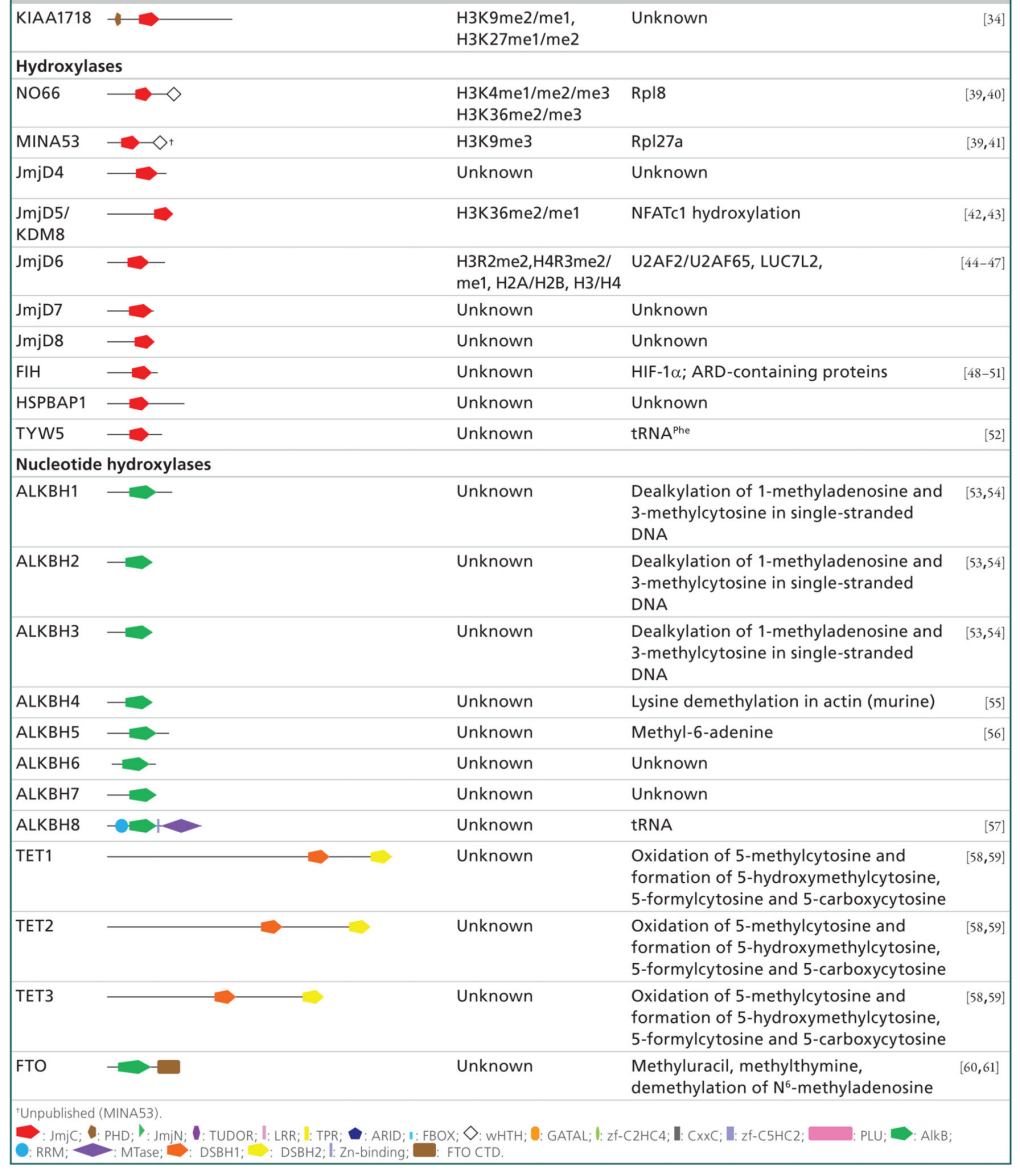

**Table 2:** Involvement of Jmj-type demethylases and other oxygenases in human physiology and disease

Member	Subfamily	Function	Human physiology and disease			
			*Development*	*Cancer*	*Inflammation*	*Other*
**KDM2**						
FBXL11; FBXL10	KDM2	H3K36me2/1 demethylase; regulation of CGI epigenetic environment	Role in reprogramming, pluripotency and maintenance of stem cell properties [97-102]	[106-108]	Regulation of NFκB activity by demethylation of Lys residue in NFkB [17]	-
**KDM3**						
JmjDI1A	KDM3A	H3K9me2/1 demethylase; coactivator of AR transcription	Sperm development (mouse knockout model) [112,113]	Overexpression in cancer cells [117] bladder, lung, hepatocellular [lis], prostate [119], colorectal [120] and renal cell carcinoma [121]	-	Obese phenotype (mouse knockout model) [112,113], transcriptional control of metabolic genes in muscle and adipose tissue [114,115]
JmjD1B	KDM3B	H3K9me2/1 demethylase; interaction with transcriptional repressors	-	Putative tumor suppressor [19,124,125]; frequently deleted in myeloid leukemias [122] and in breast cancer [123]	-	-
JmjD1C	-	Interaction with thyroid hormone receptor	-	Putative role in tumor suppression [129]	-	-
HR	-	Corepressor for several nuclear receptors (thyroid hormone, retinoic acid and vitD); interaction with HDAC	-	-	-	Mutations are associated with congenital alopecia or atrichia with papular lesions [131,138]
**KDM4**						
JmjD2A	KDM4A	H3K9/H3K36 demethylase; coregulator for nuclear receptors such as AR	Neural crest development [156]; cardiac hypertrophy [157] (murine model studies)	Role in progression of hormone responsive and hormone nonresponsive cancers (prostate, breast) [142-145]	-	-
JmjD2B	KDM4B	H3K9/H3K36 demethylase; coregulator for nuclear receptors such as AR	Mesenchymal stem cell differentiation [158] (murine model system)	Role in progression of hormone responsive and hormone nonresponsive cancers (prostate, breast) [142-145]; regulation of AR stability [146,147]; overexpressed in ER-positive primary breast cancer [151-153]	-	-
JmjD2C	KDM4C	H3K9/H3K36 demethylase; coregulator for nuclear receptors such as AR	-	Role in progression of hormone responsive and hormone nonresponsive cancers (prostate, breast) [142-145]; amplified in triple-negative (estrogen, progesterone and HER2 receptors) breast cancers [148]	-	-
JmjD2D	KDM4D	H3K9me3/2 and H1.4K26me3 demethylase	-	Interaction with p53 [161] and CAR [160]	Control of immune-cell specific enhancer elements by H3K9me3 demethylation [155]	Regulation of drug metabolism (murine) [160]
**KDM5**						
JARID1A	KDM5A	H3K4 demethylase	Control of developmental genes (e.g., HOX) [23]	NUP98/JARID1A gene fusion involved in pediatric AMKL [169], determinant of drug resistance [171]	Susceptibility gene in ankylosing spondylitis [170]	-
JARID1B	KDM5B	H3K4 demethylase	Stem cell maintenance and differentiation [172-174]	Control of cellular senescence [167]; regulation of tumor suppression/breast cancer cells [175,177]; melanoma [176]; cancer stem cell maintenance [178,179]	-	-
JARID1C	KDM5C	H3K4 demethylase	XLID [180]; ASD [182]; regulation of neuronal genes [164]	Tumor growth suppression through interaction with VHL protein [183]	-	-
JARID1D	KDM5D	H3K4 demethylase	Genomic locus is associated with AZF region [187]	Deleted in 50% of prostate cancer [188]	-	Male-specific histocompatibility antigen (H-Y) [184-186]
**KDM6**						
UTX	KDM6A	Regulation of H3K27 repressive marks, scaffolding function for chromatin complexes	Kabuki syndrome [208,209]	Inactivating somatic mutations in multiple tumor types [233]	-	-
JmjD3	KDM6B	Regulation of H3K27 repressive marks, scaffolding function for chromatin complexes	Regulation of key developmental genes [196-199]	Tumor suppressor function through activation of P16/INK4; and p14/ARF locus [226-228]. Overexpression associated with oncogenic function found in multiple tumor types [211,220-225]. Oncogenic role in EMT [229-231]. Mutations found in ALL [232]	Macrophage plasticity (mouse) [213,214]; and proinflammatory gene regulation [28,189,210]; T helper cell development [191]; possible target in SLE [215]; overexpression in vasculitis [218]	-
UTY	-	Enzymatic activity unknown, scaffolding function for chromatin complexes	-	-	-	Graft/host interaction, minor HLA-B8 antigen [235]; risk factor in cardiovascular disease [236]
**KDM7**						
KIAA1718	KDM7A	Transcriptional coactivator; removal of repressive histone marks	Regulation of neural differentiation e.g., through control of FGF4 [245]	-	-	-
PHF8	KDM7B	Transcriptional coactivator; removal of repressive histone marks	Siderius-Hamel syndrome; XLMR, cleft-lip/cleft palate [247-249]; autism spectrum disorders [251,252]	RARα coactivation in promyelocytic leukemia [243]	-	-
PHF2	KDM7C	Transcriptional coactivator; removal of repressive histone marks	-	-	Control of proinflammatory gene expression [244]	Breast cancer [246]
**Other hydroxylases**						
MINA53	-	Ribosome modification, chromatin hydroxylation/demethylation?	-	Dysregulation in various cancers [263,267,269,270,273,279]; direct target of c-myc	Allergen response [274]; TH17 cell development [275]	-
NO66	-	Ribosome modification; histone demethylation	Regulation of MSC-osteoblast differentiation [40]	Overexpression in non-small-cell lung cancer [279]	-	-
JmjD4	-	Function unknown				
JmjD5	-	Histone modification? Interaction with p53, effect on proliferation; hydroxylation of transcription factors	Growth retardation, ablation is embryonically lethal (murine) [281,282]; regulation of transcription factor half-life through hydroxylation [43]	Overexpression in leukemia and breast cancer [281]	-	Involvement in control of circadian rhythm [280]
JmjD6	-	Presumably involved in RNA splicing and/or metabolism, lysine 5-hydroxylation [45,285]	From murine models: Critical for normal development of multiple tissues such as brain, eyes, lung, kidney, liver and intestine [286-288]; ablation of its function is associated with complex cardiopulmonary malformations resembling tetralogy of Fallot [289]	Overexpression linked to poor prognosis in breast cancer [290]; lung adenocarcinoma [92]	-	-
JmjD7	-	Unknown function	-	-	-	-
JmjD8	-	Unknown activity; inhibition of proliferation and cell invasion	-	Model system for squamous cell carcinoma [292]	-	-
FIH	-	Regulation of hypoxic response	-	FIH expression is regulated by microRNA-31 in head and neck squamous cell carcinoma [257]; prognostic factor for poor overall survival in clear cell renal cell carcinoma [258]	-	FIH-null mice display metabolic changes (reduced body weight, elevated metabolic rate and resistance to effects of high-fat diet) [255,256]
HSPBAP	-	Unknown enzymatic function	-	-	-	Differential expression in intractable epilepsy (neuron, glial cells) [296,297]; neuroprotective function [295,296]
TYW5	-	tRNA hydroxylation	-	-	-	-
**Nucleotide hydroxylases**						
FTO	-	RNA N^6^-methyladenosine demethylase; demethylation of methylthymine and methyluracil	Regulation of energy homeostasis; increased postnatal lethality, growth retardation, microcephaly, psychomotor delay, dysmorphism, cleft palate, cardiac abnormalities [325]	Breast cancer [323]; colorectal cancer [324]	-	Insulin resistance, obesity [318,319]; Alzheimer’s disease [320]; cardiovascular disease [321]; end-stage renal disease [322]
TET1	-	Hydroxylation of methylcytosine to 5-hydroxymethylcytosine, 5-formylcytosine and 5-carboxycytosine	Cell differentiation, embryonic development [329]	Myeloproliferative neoplasms, CMML and AML [327-328,330]	-	-
TET2	-	Same as TET1	Cell differentiation, embryonic development [329]	Myeloproliferative neoplasms, CMML and AML [327-328,330]	-	-
TET3	-	Same as TET1	Cell differentiation, embryonic development [329]	Myeloproliferative neoplasms, CMML and AML [327-328,330]	-	-
ALKBH1	-	DNA and RNA; repair of alkylated nucleotides	-	Non-small-cell lung cancer [306]	-	-
ALKBH2	-	DNA and RNA; repair of alkylated nucleotides	-	Regulation of cell-cycle and EMT in urothelial carcinoma [305]; drug resistance in glioblastoma [307]; cancer risk in meningioma [304]	-	-
ALKBH3	-	DNA and RNA; repair of alkylated nucleotides	-	Tumor survival function in lung, pancreas, urothelial, prostate carcinoma [308-311]	-	-
ALKBH4	-	Function unknown	-	-	-	-
ALKBH5	-	RNA N^6^-methyladenosine demethylase	Impaired spermatocyte development (mouse) [56]	-	-	Genetic locus for obesity in hispanic population [331]
ALKBH6	-	Function unknown	-	-	-	-
ALKBH7	-	Function unknown	-	-	-	-
ALKBH8	-	tRNA synthesis, methyltransferase	-	Bladder cancer progression [326]	-	-

Note that several functions in physiology are inferred from animal model studies. For subfamily annotation see Figure 1.

AML: Acute myeloid leukemia; AMKL: Acute megakaryoblastic leukemia; AR: Androgen receptor; AZF: Azoospermia factor; CAR: Constitutive androstane receptor; CGI: CpG island; CMML: Chronic myelomonocytic leukemia; ER: Estrogen receptor; HDAC: Histone deacetylase; MSC: Mesenchymal stem cell; SLE: Systemic lupus erythematosus; vitD: Vitamin D; XLID: X-linked intellectual disability; XLMR: X-linked mental retardation.

## Executive summary

- Jumonji proteins were identified as transcriptional regulators in mouse knockout models, and constitute a large, evolutionarily conserved family of iron and 2-oxoglutarate-dependent oxygenase enzymes.
- The human 2-oxoglutarate family consists of over 60 members, sharing within the catalytic domains few conserved sequence motifs and a similar structural folding pattern. The enzymatically active members of this class of metalloenzymes mediate hydroxylation of several types of amino acid residues found in chromatin (e.g., methyl-lysine in histones, thereby mediating demethylation reactions), transcription factors, ribosomal and structural proteins, as well as small molecules such as lipids or metabolic intermediates. Other important functions extend to nucleotide hydroxylations, of importance in DNA repair, and in reversal of 5-methylcytosine or N6-methyladenosine modifications, which are critical for epigenetic gene regulation and regulation of RNA functions, respectively.
